# Plasma miR-150-5p in Renal Transplant Recipients with Acute Antibody-Mediated Rejection

**DOI:** 10.3390/jcm13061600

**Published:** 2024-03-11

**Authors:** Iván Zepeda-Quiroz, Carlos A. Guzmán-Martín, Mario Peña-Peña, José D. Juárez-Villa, Maria V. Soto-Abraham, Miguel A. Vázquez-Toledo, Rogelio F. Jiménez-Ortega, Bernardo Moguel-González, Horacio Osorio-Alonso, Fausto Sánchez-Muñoz, César Flores-Gama

**Affiliations:** 1 Departamento de Nefrología, Instituto Nacional de Cardiología Ignacio Chávez, México City 14080, Mexico; ivanquiroz621@gmail.com (I.Z.-Q.); daniel_00_5@hotmail.com (J.D.J.-V.); virgiliasoto@gmail.com (M.V.S.-A.); bernardomoguel@hotmail.com (B.M.-G.); 2 Departamento de Inmunología, Instituto Nacional de Cardiología Ignacio Chávez, México City 14080, Mexico; gmcarlos93@gmail.com (C.A.G.-M.); marionutricion2017@gmail.com (M.P.-P.); miguelpradyu1401@gmail.com (M.A.V.-T.); 3 Laboratorio de Genómica del Metabolismo Óseo, Instituto Nacional de Medicina Genómica, México City 14610, Mexico; rogeliofrank.jimenez@uneve.edu.mx; 4 Departamento de Ciencias de la Acupuntura, Universidad Estatal del Valle de Ecatepec, Ecatepec de Morelos 55210, Mexico; 5 Departamento de Fisiopatología Cardio Renal, Instituto Nacional de Cardiología Ignacio Chávez, México City 14080, Mexico; horace_33@yahoo.com.mx

**Keywords:** kidney transplant, miRNAs, antibody-mediated rejection, microvascular inflammation

## Abstract

**Background**: Rejection continues to be the main cause of renal graft loss. Currently, the gold standard for diagnosis is an allograft biopsy; however, because it is time-consuming, costly, and invasive, the pursuit of novel biomarkers has gained interest. Variation in the expressions of miRNAs is currently considered a probable biomarker for the diagnosis of acute rejection. This study aimed to determine whether miR-150-5p in serum is related to microvascular damage in patients with acute antibody-mediated rejection (ABMR). **Methods**: A total of 27 patients who underwent renal transplantation (RT) with and without ABMR were included in the study. We performed the quantification of hsa-miR-150-5p, hsa-miR-155, hsa-miR-21, hsa-miR-126, and hsa-miR-1 in plasma by RT-qPCR. The expressions between the groups and their correlations with the histological characteristics of the patients with ABMR were also investigated. **Results**: miR-150-5p significantly increased in the plasma of patients with rejection (*p* < 0.05), and the changes in miR-150-5p were directly correlated with microvascular inflammation in the allograft biopsies. Clinical utility was determined by ROC analysis with an area under the curve of 0.873. **Conclusions**: Our results show that the patients with RT with ABMR exhibited increased expression of miR-150-5p compared to patients without rejection, which could have clinical consequences, as well as probable utility in the diagnosis of ABMR, and bioinformatics may help in unraveling the molecular mechanisms underlying ABMR conditions.

## 1. Introduction

Renal transplantation is currently considered the ideal renal replacement therapy (RRT) for patients who reach advanced stages of chronic kidney disease (CKD), since it shows the best survival and quality of life compared to other modalities [1]. Despite improvements in surgical techniques, advances in immunology, and new immunosuppressive drugs, rejection remains the main factor conditioning renal graft loss [2].

In order to overcome this immunologic barrier, several strategies have been established, such as reducing donor–recipient incompatibility, immunosuppression therapy, and induction of tolerance [3,4]; however, rejections still continue to be a problem in the transplantation area. When prevention has failed, a timely diagnosis can improve graft survival [4]. To date, the gold standard for establishing the diagnosis of antibody-mediated rejection (ABMR) requires a histopathological evaluation of the graft obtained by biopsy [5] in addition to the presence of antibodies directed against antigens present in the donor [6]. However, biopsies are time-consuming, costly, invasive, subject to pathologist interpretation, and susceptible to sampling error. The shortcomings of current biomarkers have spawned research into new biomarkers. In recent years, microRNAs (miRNAs) have gained global recognition as potential biomarkers in a range of diseases [7]. These epigenetic regulators operate at the post-transcriptional level, impacting various cellular processes, such as proliferation, apoptosis, development, and carcinogenesis [8]. In particular miR-150, miR-155, miR-21, miR-126, and miR-1 have been implicated in regulating immune responses, inflammatory pathways, vascular function, tissue remodeling, and injury repair processes, all of which are critical in the context of transplant rejection [9,10,11,12,13]. For example, Izabella and colleagues identified specific miRNAs, such as miR-150-5p, as potential mediators of kidney fibrosis and progression in IgA nephropathy, suggesting their utility as biomarkers for predicting disease likelihood and progression [14]. In a separate investigation, conducted by Xiangnan Hao and team, treatment with LNA-anti-miR-150 exhibited effectiveness in mitigating renal interstitial fibrosis (RIF) in mice models induced with folic acid. Notably, this therapeutic approach led to improvements in fibrotic markers, such as alpha-smooth muscle actin and fibronectin. The mechanism underlying these effects involved the modulation of the SOCS1/JAK1/STAT1 pathway, resulting in a reduction in pro-inflammatory M1/M2 macrophage polarization and decreased renal infiltration [15]. While miRNAs have been explored in the context of renal transplantation, including studies on ischemia–reperfusion injury, acute kidney injury, and acute rejection, these investigations have yielded variable results [16,17]. Notably, few studies have explored the potential role of miRNAs in acute kidney rejection [18], leaving the utility of miRNAs as biomarkers largely unknown. As such, our study aimed to evaluate the expression levels of miR-150, miR-155, miR-21, miR-126, and miR-1 in the plasma of renal transplant recipients, both with and without antibody-mediated rejection, to gauge their clinical applicability and correlation with histological findings.

## 2. Materials and Methods

### 2.1. Clinical Characteristics

We conducted a cross-sectional study in all consecutive adult kidney transplant recipients with an estimated glomerular filtration rate (eGFR) ≥ 20 mL/min/1.73 m^2^ and post-transplant time > 12 weeks who underwent kidney biopsy at the Instituto Nacional de Cardiología Ignacio Chávez between May 2022 and March 2023. Recipients of combined transplants or kidney transplants after another solid organ transplant and those with active neoplasia or infection were excluded. In addition, patients were eliminated if the material collected for the histopathological analysis or measurement of miRNAs was inadequate for interpretation. The study was approved by the Institutional Review Board (23-1356) of our center and conducted in accordance with the Declaration of Helsinki. All patients provided informed consent.

### 2.2. Detection of Circulating Anti-HLA Antibodies

All included patients had a negative complement-dependent cytotoxicity crossmatch. The pre- and post-transplant anti-HLA antibodies were monitored in a single histocompatibility laboratory. Donor specific antibodies (DSAs) against HLA-A, -B, -C, -DRB1, -DRB345, -DQ, and -DP loci in the recipient serum were determined using Sigle Antigen Flow Beads Assay and measured pretransplantation and at the time of the allograft biopsy. Specificities with a mean fluorescence intensity (MFI) ≥ 1000 were considered positive. De novo DSAs (dnDSAs) were considered positive when they had not been identified pretransplantation.

### 2.3. Clinicopathological Assessment

The clinical data were collected during routine clinical follow-up. All post-transplant renal allografts clinically indicated and protocol-specified biopsies were included. In our center, protocol-specified biopsies are performed at 3 and 12 months after transplant in the absence of allograft dysfunction in retransplant patients and in those with preexisting HLA-DSAs, panel-reactive antibodies (PRAs) ≥ 30%, or when there is evidence of poor adherence.

All biopsies were analyzed by a single nephropathologist. Histological lesions were classified according to the Banff 2019 Kidney Meeting Report. An immunohistochemical C4d stain was performed on a frozen sample of tissue.

A diagnosis of rejection was established when biopsy specimens fulfilled the three Banff criteria for ABMR. Control biopsies were those considered as normal or with nonspecific changes according to the Banff 2019 classification [19].

### 2.4. miRNA Determination

Blood samples for the measurement of miRNAs were obtained at the time of allograft biopsy and stored for later analysis, blinding for the histopathological report.

The quantification of miRNAs was performed as follows: 200 μL of plasma was used for the detection of miRNAs using reagents and protocols for the (manufactured by Applied Biosystems, Bedford, MA, USA) TaqMan MicroRNA Reverse Transcription Kit and TaqMan MicroRNA Assay (Applied Biosystems). The miRNA assays used are as follows: manufactured by Applied Biosystems, Bedford, MA, USA) hsa-miR-150, assay ID: 000473, catalog number: 4427975; hsa-miR-155, assay ID: 002623, catalog number: 4427975; hsa-miR-21, assay ID: 000397, catalog number: 4427975; hsa-miR-126, assay ID: 002228, catalog number: 4427975; and hsa-miR-1, assay ID: 475545, catalog number: 4440886. The RT reactions were performed with custom stem-loop primers (Applied Biosystems) specific for the corresponding miRNA mature sequence obtained from miRbase (available at: http://www.miRBase.org (accessed on 3 June 2013)). The amplification reactions were performed using the CFX96 real-time PCR system (BIORAD). Quantitative RT-PCR data were analyzed using the comparative threshold cycle (Ct) method, with hsa-miR-16 as the endogenous reference for plasma. The relative expressions of the miRNAs were obtained using the following arithmetic formula: 2^−ΔΔCt^.

### 2.5. Statistical Analysis

The statistical analysis was performed using the Statistical Package for Social Sciences (SPSS v26, developed by IBM, Armonk, NY, USA). The normality was determined with the Shapiro–Wilk test. A comparison of the quantitative variables between the two groups was carried out with the Mann–Whitney U test, and the comparison among more than two groups was carried out using the Kruskal–Wallis test. To test the discriminative value of miR-150 among the groups of interest, we performed an area under the curve (AUC) analysis. The optimal cutoff of miR-150 was determined by the Youden’s Index. The qualitative variables were compared using the chi-square test. Finally, Spearman’s method was used to search for correlations among the quantitative variables. A *p*-value less than 0.05 was considered significant.

### 2.6. Bioinformatic Analysis

#### 2.6.1. Prediction of miRNA Target Genes

To predict the potential target genes of the miRNA hsa-miR-150, different algorithms based on computational methods were used, whose function is to identify the nucleotide pairing between the seed region of a miRNA (2–7nt) and the 3’UTR region of its mRNA. Databases such as TargetScan are based on the accessibility of the binding site of the target gene, while databases such as miRanda use software based on the thermodynamic properties of the miRNA allowing for the filtering of the binding sites in the “seed” region in the 3’UTR region of the mRNA, whereas other software applications use machine learning based on the parameterization of biological data and other predicted features [20,21,22]. The databases used were (Developed by Wong lab, Boston, MA, USA) miRDB v 6.0, accessed on 21 February 2024 (http://mirdb.org/miRDB/), (Developed by Bioinformatics, Saarland, Germany) miRWalk v 3.0 (accessed on 21 February 2024) (http://mirwalk.umm.uni-heidelberg.de/), (Developed by Massachusetts Institute of Technology (MIT), Cambridge, MA, USA) TargetScan v 7.2 (accessed on 21 February 2024) (http://www.targetscan.org/vert_80/), and (Developed at the Bioinformatics and Biological Computing unit, Rehovot, Israel) PITA v5 0.0 (accessed on 21 February 2024) (https://tools4mirs.org/software/target_prediction/pita/). Identified target genes were selected if they were present in at least 3 of the selected databases, and the predicted target genes for each miRNA were merged into a single list for further comparative analysis.

#### 2.6.2. Expression Microarray Analysis

To select genes associated with kidney transplantation that were targets of miR-150, data in the CEL format from the GeneChip Human Genome U133 plus 2.0 microarray of the Affymetrix platform were analyzed, which were obtained from Gene Expression Omnibus (https://www.ncbi.nlm.nih.gov/geo/ (accessed on 21 February 2024)) (Developed by National Center for Biotechnology Information NCBI, Bethesda, MD, USA) with accession number GSE36059. In this work, the expression profiles of genes associated with histological rejection mediated by T cells in kidney transplant biopsies were analyzed; however, for our analysis we only extracted data from the ABMR and nonrejection groups. They have limited reproducibility due to nonspecific lesions that use arbitrary rules that are subject to different interpretations [23].

#### 2.6.3. Data Processing and Differentially Expressed Genes (GDEs)

The differential expression analysis was performed using original files that were obtained in the CEL format (GSE36059) and processed into expression values through the “robust multiarray average” (RMA) method in the “R” environment with the “R” packages “Affy”. The probe-level data were later transformed using R-BiocManager, followed by background correction and data normalization. The cut-off criteria for choosing overexpressed genes included fold-change values < −0.5 and >0.5 in addition to a “false discovery rate” (FDR) value < 0.05.

#### 2.6.4. Selection of Potential Candidate Genes

This group of shared genes was analyzed using ShinyGO Enrichment Analysis + more software v0.741 (http://bioinformatics.sdstate.edu/go74/ (accessed on 21 February 2024)) (Developed by South Dakota State University, Brookings, SD, USA) and in the species Homo sapiens to obtain enrichment pathways from the KEGG database, showing correlations among the enrichment pathways and, thus, generating an interaction network among the most significant biological processes. Using the STRING-KEGG Pathway tool (https://string-db.org/ (accessed on 21 February 2024)), the signaling pathways involved in kidney transplant rejection were obtained and selected. This selected group was used to develop an interaction network between miR-150 and the target genes involved using Cytoscape v3.9.1 software.

## 3. Results

Of the 30 renal allograft biopsies on the 30 included patients, 3 were eliminated due to a lack of data, and, in total, 27 patients were considered in the statistical analysis. Among the sociodemographic characteristics of the population studied, 51.9% were women, with a median age of 42 years (IQR: 32–51). In most patients, the cause of CKD was unknown (59.3%), most underwent living-donor transplantation (51.8%), and the median time between transplantation and biopsy was 80.5 months (Table 1). The average level of creatinine at the time of biopsy was 1.5 mg/dL; 70% of the biopsies were performed because of graft dysfunction, and the rest were protocolized. A total of 15 biopsies were classified as ABMRs and 12 as normal or with minimal nonspecific alterations (Table 1).

It was found that the group of patients with ABMR were younger, more frequently documented poor therapeutic adherence, had biopsies that were performed at a longer post-transplant time, and had a higher prevalence of pre- and post-transplant DSAs compared to the group without ABMR, with no differences in the type of donor, induction or maintenance therapy, serum creatinine levels, or the reason for the biopsy (indication vs. protocol) between the groups.

### 3.1. Histopathological Features

From each renal allograft biopsy, the histological findings are reported according to the Banff 2019 Kidney Meeting Report (Table 2).

More frequently, the group of patients with ABMR presented glomerular (100% vs. 58.3%, *p* = 0.01), mesangial (100% vs. 58.3%, *p* = 0.01), and peritubular capillary (100% vs. 8.3%, *p* < 0.001) inflammation. Similarly, tubular (53.3% vs. 8.3%, *p* = 0.02) and interstitial (60% vs. 0%, *p* = 0.001) inflammation occurred more frequently in the ABMR group. For chronic lesions, only glomerular capillary duplication (40% vs. 0%, *p* = 0.02) and chronic vascular changes (53.3% vs. 25%, *p* = 0.14) were different between the groups, with a higher degree of involvement in the ABMR group.

### 3.2. Differential Expression of miR-150-5p in Plasma Samples in Patients with ABMR and Performance of Circulating miR-150-5p in the Diagnosis of ABMR

**Figure 1:**
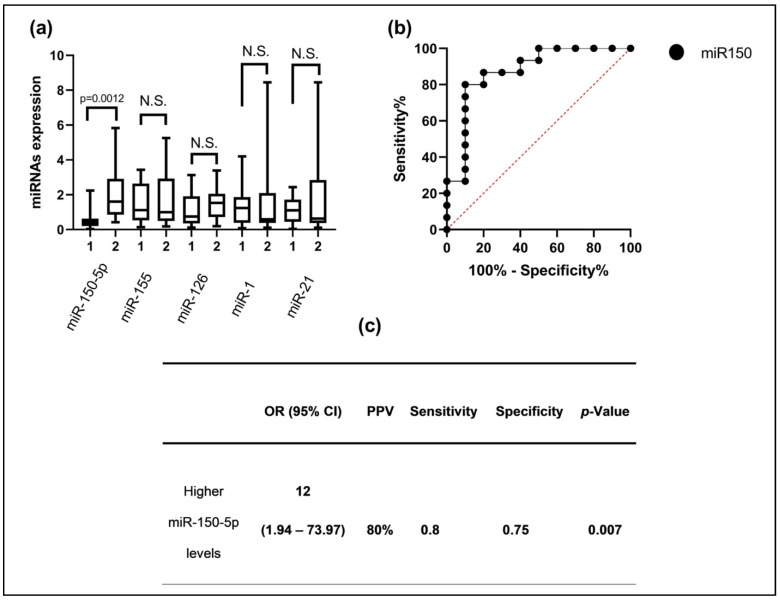
(**a**) The hsa-miR-150-5p, hsa-miR-155, hsa-miR-21, hsa-miR-126, and hsa-miR-1 expression levels between patients with ABMR and without transplant rejection; (**b**) ROC analysis of the area under the miR-150-5p curve to discriminate between the two groups of interest; (**c**) patients with higher levels of miR-150-5p have an increased risk of suffering from ABMR. 1 = Without rejection; 2 = ABMR; N.S. = nonsignificant.

First, we analyzed the plasma expression levels of hsa-miR-150-5p, hsa-miR-155, hsa-miR-21, hsa-miR-126, and hsa-miR-1. Interestingly, we only found differences for hsa-miR-150-5p, specifically, a higher level of expression of miR-150-5p in the group with ABMR (medians: 1.60 vs. 0.42) (*p* < 0.0012) (Figure 1a). Then, to assess the predictive ability of the validated miR-150-5p for ABMR risk, an AUC-ROC curve for analysis was constructed comparing the cases of ABMR with the patients who did not experience rejection. This was performed using data obtained with the validation reverse transcription polymerase chain reaction technique. The results reveal that miR-150-5p demonstrated an effective discriminatory ability, with an AUC of 0.87 (95% CI: 0.7195 to 1.00). The optimal expression threshold for predicting ABMR was set at >0.96, which provided a sensitivity of 63.16% and a specificity of 89.47% (Figure 1b). Finally, using the Youden’s Index, we established an optimal cut-off point for miR-150-5p, and to rely on this cut-off point, we divided all patients into two groups to perform the Fisher test with a cross-table analysis. Interestingly, we found a higher risk for ABMR in the high-level miR-150-5p group (OR: 12); in addition, we calculated the PPV, specificity, and sensitivity in this cross-table analysis (Figure 1c).

**Figure 2:**
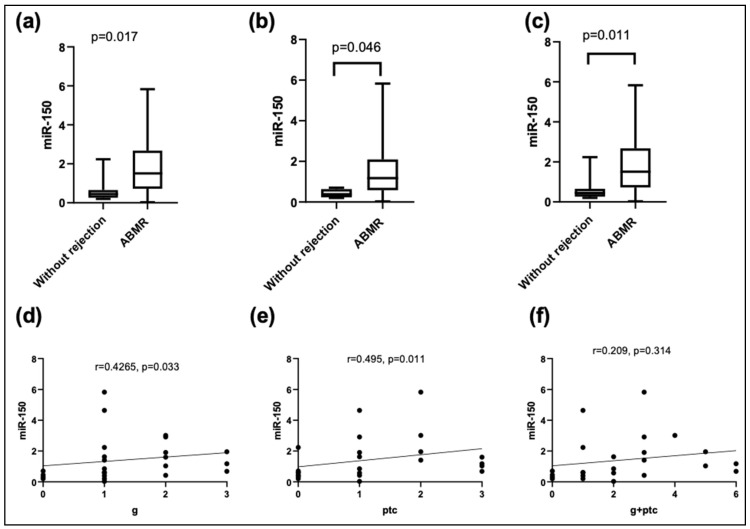
Relative expression levels of miR-150-5p in plasma according to the (**a**) presence of microvascular inflammation (mvi); (**b**) glomerulitis (g); (**c**) peritubular capillaritis (ptc). Correlation analysis using Kendall’s Tau-b between miR-150-5p and histological characteristics: (**d**) g; (**e**) ptc; (**f**) g + ptc.

Then, when comparing the plasma expressions of miR-150-5p with the histological findings, higher levels were found in the patients with mvi and ptc. In addition, the expressions of plasma miR-150-5p showed a moderate positive correlation with the degree of inflammation in both glomerular and peritubular capillaries but not with other histological lesions, whether acute or chronic (Figure 2).

**Figure 3:**
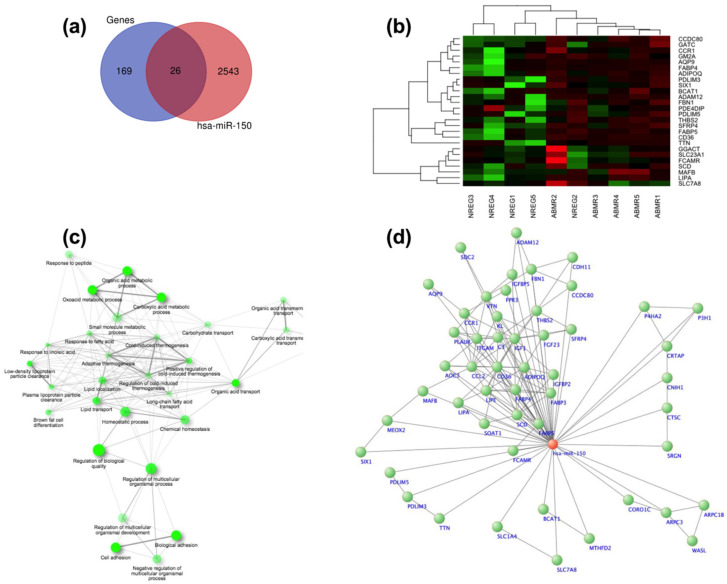
Bioinformatic analysis: (**a**) Venn diagram illustrating the mRNA target genes identified from databases and microarray expression analysis; (**b**) heatmap illustrating the genes exhibiting differential expression between patients without rejection and those with antibody-mediated rejection (ABMR); (**c**) by conducting the enrichment analysis using the KCGG, we generated an interaction network depicting the main signaling pathways associated with the dysregulated genes; (**d**) we constructed an interaction network involving genes from the key signaling pathways associated with renal transplantation and their associations with miR-150.

We conducted comprehensive bioinformatic analyses, as outlined in Section 2. Initially, we utilized predictive algorithms to identify mRNA target genes for miR-150, resulting in a list of 2544 potential targets obtained from various databases. Subsequently, we intersected this list with the data from the differential expression analysis of the microarrays linked to miR-150, yielding 26 shared genes between the two analyses (refer to Figure 3a,b). These shared genes were further analyzed using ShinyGO v0.741 to explore the enrichment pathways from the KEGG database, revealing significant correlations among the pathways. From this analysis, we identified several key signaling pathways, including those related to biological and cell adhesion, vascular transport, carbohydrate transport, and lipid metabolism, among others, which are known to play crucial roles in kidney transplant rejection (see Figure 3c). To visualize the interaction between miR-150 and its target genes involved in these pathways, we developed an interaction network using Cyto-scape v3.9.1 software (see Figure 3d). 

## 4. Discussion

The present study evaluated the expression levels of miR-150 in the plasma of renal-transplant-recipient patients, with and without ABMR in renal biopsies by indication and protocolized by RTq-PCR. We found higher levels of miR-150 in patients with ABMR. In addition, higher levels of microvascular inflammation, both g and ptc, were associated with a higher plasma miR-150-5p level but not for acute or chronic lesions in other renal compartments.

miR-150-5p is associated with immune regulatory functions, proliferation, activation, and apoptosis of B and T lymphocytes, and it is selectively expressed in lymph nodes, spleen, and mature T and B cells. In addition, it has been observed that miR-150-5p could be a suppressor of angiopoietin 2 generation, playing key roles in the resolution of vascular injury and reduction in mortality resulting from sepsis [9], as well as its overexpression in mice with lupus nephritis and its prognostic role in patients with IgA nephropathy [14]. It is through these expressions in T and B lymphocytes, as well as their roles in vascular repair, main cells, and immune targets in ABMR, that miR-150-5p may play an important role as a biomarker.

miR-150-5p has been studied in patients with acute rejection, showing higher expression in patients with acute cellular rejection compared to patients without rejection, whereby miRNA determination was performed through RNA extraction in kerosene blocks; in our case, it was not possible to explore its role in T-cell-mediated rejection, since, because of the baseline immunological risk of our population and the current immunosuppressive therapy, only one case of cellular rejection was registered [13]. On the other hand, Alfaro et al. observed a decrease in the expression of miR-150-5p in patients with acute rejection independently of the type of rejection, whereby they were obtained by isolation of peripheral blood leukocytes, observing that CD4+ cells decrease the levels of intracellular miR-150 in patients with acute rejection [18]. A study by Candia et al. (2013) demonstrated that after activation of CD4+ cells, there is a decrease in miR-150 at the intracellular level that is correlated with the increase in the plasma [24]. This premise could explain the differences between our study and what has previously been reported, whereby patients with ABMR have increased serum miR-150-5p levels but decreased intracellular ones as a result of CD4+ cell activation. Our finding is compatible with what has been reported in murine pancreatic transplantation models, for which an elevation of plasma miR-150-5p has been documented, associated with in-graft rejection and β-cell destruction [25].

Regarding studies that have explored the usefulness of miR-150-5p levels as a biomarker in renal transplant patients, the results are still controversial. A study published in 2017 showed that increased plasma miR-150-5p may be biomarker in renal transplantation [26], whereas, in 2022, it was reported that only in women did miR-150-5p decrease in cases of post-transplant dysfunction [27].

Among our findings, we report an increased miR-150-5p expression in graft biopsies showing microvascular inflammation, both ptc and g. Modifications in the expression of miR-150-5p in vascular lesions have been documented in patients with sepsis, being considered as a new suppressor of angiopoietin 2 (Ang2) generation, playing key roles in the resolution of vascular lesions and the reduction in mortality in these patients [28].

It is notable that the difference in expression is less marked in cases of ptc and g of grade 3. Despite this, it is important to note that this expression remains consistently higher in comparison with patients without ABMR, which could be related to vascular damage being too important to condition the use of serum miR-150-5p for the search to the resolution to endothelial damage. Similarly, miR-150-5p has been proposed as a possible functional mediator of renal fibrosis in patients with IgA nephropathy, which could predict the risk of progression in this context; the presence of miR-150-5p in patients with ABMR could be conditioning the regulation of fibrosis [14], as was demonstrated in an experimental study in mice in which miR-150-5p inhibition had the potential to prevent tubulointerstitial fibrosis by suppressing the SOCS1/JAK/STAT pathway, also demonstrating that increased miR-150-5p expression in renal tissue conditions greater fibrosis. Nevertheless, our study’s design does not substantiate this claim. Larger and more comprehensive studies addressing this issue are imperative to obtain conclusive evidence [29].

To the best of our knowledge, our study is the first to perform a correlation analysis between miR-150-5p expression levels and histopathological findings in renal allografts, showing that plasma elevation is higher in those who present microvascular inflammation, exploring both the degree of fibrosis and acute over chronic inflammation, without finding significant differences in miR-150-5p expression levels. Several studies have suggested that miRNA expression levels may be affected by the glomerular filtration rate (GFR), leading to an accumulation of these molecules in serum due to decreased clearance [30]. However, in our study, no correlation was observed between miR-150-5p levels and creatinine levels, suggesting that this molecule is not influenced by renal function and its determination is not altered by it.

The increased expression of miR-150-5p in patients with ABMR could be associated with a set of target genes, such as the *MBD6* gene which translates the MBD6 protein, which binds to methylated DNA and participates in aging, proliferation, and cell survival processes [31]. Considering that the modification of its serum levels could be an important response to attenuate a renal rejection event, it is important, however, to highlight that the different studies that have attempted to validate miR-150-5p are not homogenous in terms of the type of rejection, as well as the miRNA extraction site. Taking into account that our study intends to propose miR-150-5p as a biomarker, it is important to note that the extraction of this miRNA was from plasma, in which its elevation is evidenced in contrast to the study by Alfaro and colleagues [18]. This variation is compatible with CD4+ activation.

The bioinformatic analysis conducted in this study provided valuable insight into the potential mechanisms underlying miR-150 involvement in kidney transplant rejection. By identifying mRNA target genes and elucidating enrichment pathways, we gained a deeper understanding of the biological processes influenced by miR-150 dysregulation. The significant correlations observed in key signaling pathways, such as those related to biological and cell adhesion, vascular transport, and lipid metabolism, underscore the multifaceted roles of miR-150 in modulating immune responses and tissue homeostasis. These findings suggest that miR-150 may exert its effects on transplant rejection through intricate regulatory networks involving various cellular processes. Moreover, the interaction network constructed using Cyto-scape v3.9.1 software provides a visual representation of the complex interplay between miR-150 and its target genes, offering insights into potential therapeutic targets for mitigating transplant rejection. In this context, it is worth noting that several studies have harnessed bioinformatic analyses to elucidate various pathological conditions via gene–miRNA interactions [32]. These advanced technologies hold substantial promise for shaping the development of novel drug targets in the future.

## 5. Conclusions

In conclusion, the results of this study show that patients with RT who present ABMR have an increased serum miR-150-5p expression in contrast to transplants without rejection; with these findings, we consider that miR-150-5p could have a potential to diagnose ABMR. Future studies are needed to corroborate these results and explore its performance in RT recipients with other pathologies. Finally, our findings emphasize the power of bioinformatic analyses in unraveling the molecular mechanisms underlying ABMR conditions. This underscores the significance of computational tools in studying gene–miRNAs interactions and their role in disease progression.

## 6. Limitations and Strengths

This study has some limitations that need to be addressed. Firstly, the relatively small sample size of 27 renal transplant recipients may limit the generalizability of our findings to broader patient populations. Moreover, resource limitations constrained the scope of experimental analyses, hindering comprehensive investigations into the biological mechanisms underlying miR-150-5p involvement in transplant rejection. It will be essential to tackle these constraints in future research initiatives to improve the clinical significance of miRNA-based biomarkers and further our comprehension of kidney transplant rejection mechanisms. The utilization of human clinical samples provides valuable insight into the expression patterns of miR-150-5p in renal transplant recipients, enhancing the translational relevance of the findings. The comprehensive analysis of histopathological features alongside miRNA expression levels contributes to a holistic understanding of the molecular and pathological aspects of antibody-mediated rejection. Moreover, further elucidating the specific biological pathways and mechanisms involving miR-150-5p in transplant rejection through advanced experimental techniques and bioinformatics analyses holds significant potential to enhance diagnostic and therapeutic strategies in renal transplantation.

## Figures and Tables

**Table 1:** Patient characterization. The qualitative variables are presented as frequencies and percentages and the quantitative as medians and interquartile ranges or the mean and standard deviation according to the distribution. BMI: body mass index; DSAs: donor-specific antibodies; IL-2R-inh: interleukin-2 receptor inhibitor; MGN: membranous glomerulonephritis; MPD: methylprednisolone; PRA: panel-reactive antibodies.

Variable	All Patients (n = 27)	Without Rejection (n = 12)	ABMR (n = 15)	*p*-Value
Female	14 (51.9)	8 (66.7)	6 (40)	0.16
Age	42 (32–51)	51 (37–59)	34 (31–42)	0.03
BMI (kg/m^2^)	26 ± 5	26 ± 6	25.8 ± 4	0.84
Etiology				0.12
Unknown	16 (59.3)	5 (41.6)	11 (73.3)	
MGN	9 (33.3)	5 (41.7)	4 (26.7)	
Diabetes	2 (7.4)	2 (16.7)	0	
Time post-transplantation (months)	80.5 (16–112)	37.8 (10.2–102)	85 (37.5–112)	0.22
Deceased donor	13 (48.2)	7 (58.3)	6 (40)	0.29
Dysfunction	19 (70.4)	8 (66.7)	11 (73.3)	0.52
Creatinine (mg/dL)	1.5 (0.9–2.1)	1.1 (0.9–2.0)	1.7 (1.2–2.1)	0.33
Induction				1.0
MPD	2 (7.4)	1 (8.3)	1 (6.7)	
IL-2R-inh	15 (55.6)	7 (58.3)	8 (53.3)	
Thymoglobulin	10 (37)	4 (33.3)	6 (40)	
Poor adherence	8 (29.6)	2 (16.7)	6 (40)	0.19
Allosensitization	17 (63)	6 (50)	11 (73.3)	0.2
PRA I	1 (0–5)	1 (0–2)	3 (0–10)	0.34
PRA II	2 (1–6)	2 (0.5–6)	2 (1–44)	0.57
DSAs pretransplantation	9(33.3)	2 (16.7)	7 (46.7)	0.15
DSAs post-transplantation	20 (74.1)	6 (50)	15 (100)	0.003

**Table 2:** Histological characteristics according to the Banff 2019 Kidney Meeting Report.

	All Patients (n = 27)	Without Rejection (n = 12)	ABMR (n = 15)	*p*-Value
Sclerosis	0 (0–25)	0 (0–21)	9 (0–31)	0.55
g > 0	22 (81.5)	7 (58.3)	15 (100)	0.01
ptc > 0	16 (59.3)	1 (8.3)	15 (100)	<0.001
mm > 0	22 (81.5)	7 (58.3)	15 (100)	0.01
i > 0	9 (33.3)	0	9 (60)	0.001
t > 0	9 (33.3)	1 (8.3)	8 (53.3)	0.02
v > 0	3 (11.1)	0	3 (20)	0.16
cg > 0	6 (22.2)	0	6 (40)	0.02
ci > 0	22 (81.5)	9 (75)	13 (86.7)	0.39
ct > 0	22 (81.5)	9 (75)	13 (86.7)	0.39
cv > 0	11 (40.7)	3 (25)	8 (53.3)	0.14
i-IFTA > 0	14 (52.9)	5 (41.7)	9 (60)	0.29
c4d > 0	9 (33.3)	5 (41.7)	4 (26.7)	0.34

glomerulitis (g), peritubular capillaritis (ptc), mesangial matrix increase (mm), transplant glomerulopathy (cg), in addition to tubular changes such as tubulitis (t), and tubular atrophy (ct), while for interstitium interstitial inflammation (i) and interstitial fibrosis (ci). Tubulointerstitial inflammation (ti) and interstitial fibrosis with tubular atrophy (i-IFTA). As for the vessels, arteritis of the intima (v) and fibrous thickening of the arterial intima (cv).

## Data Availability

The raw data are available directly from the corresponding authors upon reasonable request.
